# Functional Analysis of Sheep POU2F3 Isoforms

**DOI:** 10.1007/s10528-019-09945-x

**Published:** 2019-12-31

**Authors:** Guang-Wei Ma, Yan-Kai Chu, Hua Yang, Xiao-Hong Yan, En-Guang Rong, Hui Li, Ning Wang

**Affiliations:** 1 College of Animal Science and Technology, Northeast Agricultural University, Harbin, 150030 People’s Republic of China; 2 Key Laboratory of Chicken Genetics and Breedings, Ministry of Agriculture and Rural Affairs, Harbin, 150030 People’s Republic of China; 3 Institute of Animal Husbandry and Veterinary Medicine, Xinjiang Academy of Agricultural and Reclamation Science, Shihezi, 832000 People’s Republic of China; 4 State Key Laboratory of Agrobiotechnology, China Agricultural University, Beijing, 100193 People’s Republic of China

**Keywords:** POU2F3, Isoforms, Cell proliferation, Promoter activity, Sheep

## Abstract

POU domain class 2 transcription factor 3 (POU2F3) plays an important role in keratinocyte proliferation and differentiation. Our previous study identified four sheep *POU2F3* transcript variants (*POU2F3-1*, *POU2F3-2*, *POU2F3-3*, and *POU2F3-4*), encoding three POU2F3 protein isoforms (POU2F3-1, POU2F3-2, and POU2F3-3). However, the functional differences among the three POU2F3 isoforms remain unknown. The objective of this study was to determine the tissue expression pattern of the four *POU2F3* transcript variants in sheep and to investigate the functional differences in cell proliferation among the three POU2F3 isoforms. Quantitative RT-PCR analysis showed that the four *POU2F3* transcripts were ubiquitously expressed in all tested adult sheep tissues, and *POU2F3-1* exhibited higher expression level than the other three *POU2F3* transcript variants in skin (*P* < 0.05). Cell proliferation assay showed that overexpression of any one of the three POU2F3 isoforms significantly inhibited the proliferation of sheep fetal fibroblasts and HaCaT cells at 48 and 72 h after transfection (*P* < 0.05). POU2F3-3 had less inhibitory effect on cell proliferation than POU2F3-1 and POU2F3-2 (*P* < 0.05), and POU2F3-1 and POU2F3-2 had similar inhibitory effects (*P* > 0.05). Dual luciferase reporter assays demonstrated that overexpression of any one of the three POU2F3 isoforms significantly inhibited the promoter activities of keratin 14 (*KRT14*) and matrix metalloproteinase 19 (*MMP19*) genes (*P* < 0.05). POU2F3-3 had less inhibitory effect on the promoter activities of *KRT14* and *MMP19* genes than POU2F3-1 and POU2F3-2 (*P* < 0.05), and POU2F3-1 and POU2F3-2 had similar inhibitory effects (*P* > 0.05). These results suggest three sheep POU2F3 isoforms have similar functional effects, but to a different extent.

**Electronic supplementary material:**

The online version of this article (10.1007/s10528-019-09945-x) contains supplementary material, which is available to authorized users.

## Introduction

POU domain class 2 transcription factor 3 (POU2F3), a POU domain transcription factor also known as Skn-1a or Oct-11, is predominantly expressed in the suprabasal layers of the epidermis (Goldsborough et al. 1993; Hildesheim et al. 2001). POU2F3 trans-activates the expression of keratinocyte differentiation marker genes, such as keratin 10 gene (*KRT10*) (Byrne et al. 1994) and small proline-rich protein 2A gene (*SPRR2A*) (Steinert and Marekov 1995), indicating that POU2F3 promotes keratinocyte differentiation (Takemoto et al. 2010). However, a previous study demonstrated that human POU2F3 (*hPOU2F3*) mainly promoted keratinocyte proliferation and secondarily enhanced keratinocyte differentiation (Hildesheim et al. 2001). In addition, a knockout study showed that POU2F3-knockout mice had higher proliferation rate of epidermal keratinocytes adjacent to the wound edge compared with the littermate controls (Andersen et al. 1997). A colony inhibition assay showed that human POU2F3 overexpression inhibited the proliferation of cervical cancer cell lines of epithelial origin (Yutaka et al. 2004). Keratin 14 gene (*KRT14*) and matrix metalloproteinase 19 genes (*MMP19*) are highly expressed in mitotically active epithelial basal cells and promote cell proliferation (Alam et al. 2011; Beck et al. 2007). POU2F3 overexpression inhibited the expression and promoter activity of the *KRT14* gene in normal human epidermal keratinocytes (Sugihara et al. 2001) and of *MMP19* gene in HaCaT cells (Beck et al. 2007; Sadowski et al. 2003).

Similar to other *POU* genes such as *Oct-1*, *Oct-2*, *Brn-3*, and *Pit-1* (Ryan and Rosenfeld 1997), the *POU2F3* gene can generate various transcript variants, with different expression patterns and functions. In rat epidermis, the *Skn-1* gene generates two transcript variants with different functions, mSkn-1a and mSkn-1i (Andersen and Rosenfeld 1993). In human keratinocytes, *Skn-1* gene produces three different transcript variants (*hSkn-1a*, *hSkn-1d1* and *hSkn-1d2*) due to the alternative promoters, resulting in three proteins with different N-termini (hSkn-1a, hSkn-1d1 and hSkn-1d2) (Cabral et al. 2003). In our previous study, we identified four *POU2F3* transcript variants: *POU2F3-1*, *POU2F3-2*, *POU2F3-3*, and *POU2F3-4* (GenBank accession nos. JX184905, JX184906, JX184907, and JX184908), due to alternative splicing in Chinese Merino sheep, producing three protein isoforms with different N-termini (POU2F3-1, POU2F3-2, and POU2F3-3) (Rong et al. 2013). POU2F3-1 is the full-length POU2F3 (aa 1-435), POU2F3-2 (aa 34-435) lacks partial N-terminal 33 amino acids, and POU2F3-3 (aa 209-435) lacks the complete N-terminal and partial POU-specific domain (Rong et al. 2013). To date, the functional differences among the three POU2F3 isoforms remain unknown. In this study, we detected the tissue expression patterns of the four *POU2F3* transcript variants in sheep and compared the effects of the three POU2F3 isoforms on the proliferation of sheep fetal fibroblasts (SFFs) and HaCaT cells, and on the promoter activities of *KRT14* and *MMP19* genes.

## Materials and Methods

### Ethics Statement

All animal work was carried out according to the guidelines for the care and use of experimental animals established by the Ministry of Science and Technology of the People’s Republic of China (Approval number: 2006-398) and approved by the Laboratory Animal Management Committee of Northeast Agricultural University.

### Animals and Tissue Collection

Three rams from the superfine wool strain of Chinese Merino sheep (Junken Type), bred by the Xinjiang Academy of Agricultural and Reclamation Science were used for *POU2F3* gene expression analysis. The 240-day-old sheep were slaughtered, and heart, liver, spleen, kidney, rumen, small intestine, skeletal muscle, and body side skin samples were collected. All collected tissue samples were snap-frozen in liquid nitrogen and stored at − 80 ℃ for further analysis. The ear notch samples were collected during shearing. All sheep were kept in the same environment with free access to feed and water.

### Cell Culture

HEK293 and HaCaT cells were purchased from the China Center for Type Culture Collection, and cultured in DMEM (Gibco). Sheep fetal fibroblasts (SFFs) as a kind gift from Dr. Tie-Zhu An, Northeast Forestry University, Harbin, were grown in DMEM-F12 (Gibco). Both DMEM and DMEM-F12 were supplemented with 10% FBS (Gibco) and 1% streptomycin/penicillin (Gibco). All cells were cultured in a humid environment with 5% CO_2_ in the air at 37 ℃.

### RNA Extraction and Quantitative RT-PCR Assay

Total RNA of the frozen tissues or HaCaT cells was isolated using Trizol reagent (Invitrogen) according to the manufacturer's instructions, and RNA quality was assessed by denaturing formaldehyde agarose gel electrophoresis. Reverse transcription of total RNA was performed using the Promega Improm-II reverse transcription System (Promega) according to the manufacturer's instructions. Quantitative RT-PCR was carried out using SYBR Green PCR reagents on the 7500 real-time PCR system (Applied Biosystems) according to the manufacturer's instructions. Quantitative RT-PCR was performed in triplicate for each sample. Sheep *GAPDH* or human *GAPDH* was used as the internal reference for the normalization of gene expression, and the relative mRNA expression was analyzed using $2^{{ - \Delta \Delta C_{{\text{t}}} }}$ (Livak and Schmittgen 2001). The primers used for quantitative RT-PCR are shown in Table 1.

**Table 1:** Primers used for quantitative RT-PCR

Gene name	Primer pair	Primer sequence (5′–3′)	Product (bp)
*POU2F3-1*	POU2F3-1-P1-F	ATGGCTTAGATTTCAACAGG	241
	POU2F3-1-P1-R	GGCTGCAGACCTTGCT	
*POU2F3-2*	POU2F3-2-P1-F	AGCCAGGTGGAGACAGATTAAAACT	255
	POU2F3-2-P1-R	TGCGGAAAGGGGAGAAGGTT	
*POU2F3-3*	POU2F3-3-P1-F	TCAACAGGCAGGTCTGCAGC	148
	POU2F3-3-P1-R	TGCTTCCAGATGGGGTTCTAAAG	
*POU2F3-4*	POU2F3-4-P1-F	GGCAGCAAGGCAGTTG	240
	POU2F3-4-P1-R	GCTGAAGTCGTTTCCATACA	
*Ki67*	Ki67-P1-F	AGGATGGAAGCAAGTCACCTGGAT	129
	Ki67-P1-R	CTTCTGAACGGGGACTGGAATCTT	
*PCNA*	PCNA-P1-F	CGTCTCATGTCTCCTTGGTGCA	104
	PCNA-P1-R	GGACATGCTGGTGAGGTTCA	
*GAPDH*	GAPDH-P1-F	CTGACCTGCCGCCTGGAGAAA	149
	GAPDH-P1-R	GTAGAAGAGTGAGTGTCGCTGTT	

### Plasmid Construction

The full-length CDS of the three sheep POU2F3 isoforms were amplified from the recombinant plasmids (pEASY-T1-POU2F3-1, pEASY-T1-POU2F3-2, pEASY-T1-POU2F3-3), respectively, which were previously generated in our laboratory (Rong et al. 2013), and cloned into the *EcoR* I-*Xho* I sites of the pCMV-Myc vector (Clontech), named pCMV-Myc-POU2F3-1, pCMV-Myc-POU2F3-2, and pCMV-Myc-POU2F3-3, respectively. For the promoter reporter plasmid construction, the 662-bp promoter fragment (− 699 to − 38 relative to the start codon ATG of sheep *KRT14* gene) (Sugihara et al. 2001) and the 519-bp promoter fragment (− 542 to − 24 relative to the start codon ATG of sheep *MMP19* gene) (IM et al. 2007) were amplified from the sheep genomic DNA (50 ng/μL), and subsequently cloned into the KpnI–HindIII sites of the pGL3-basic vector (Promega), named pGL3-basic-pKRT14 (− 699/ − 38) and pGL3-basic-pMMP19 (− 542/ − 24), respectively. All the constructions were confirmed by sequencing. The primers used for plasmid construction are listed in Table 2.

**Table 2:** Primers used for plasmid construction

Gene name	Primer pair	Primer sequence (5′–3′)	Product (bp)
*POU2F3-1*	POU2F3-1-P2-F	CCG*GAATTC*^a^ TGGTGAATCTGGAACCCATGCA	1305
	POU2F3-1-P2-R	G*CTCGAG*^b^ TCAGTGGAGGTACGTGGGA	
*POU2F3-2*	POU2F3-2-P2-F	CCG*GAATTC*TGCTCAAAGTGCTCTCAGCC	1206
	POU2F3-2-P2-R	G*CTCGAG*TCAGTGGAGGTACGTGGGATGACT	
*POU2F3-3*	POU2F3-3-P2-F	CCG*GAATTC*TGGGAAAGCTGTATGGAAACGAC	681
	POU2F3-3-P2-R	G*CTCGAG*TCAGTGGAGGTACGTGGGATGACT	
*KRT14*	KRT14-P1-F	G*GGTACC*^c^ CTAGGGTCGGTGCTAAGGGCT	662
	KRT14-P1-R	C*AAGCTT*^d^ GTCGGCAGGGAAGAGAAAGGT	
*MMP19*	MMP19-P1-F	G*GGTACC*CCACCCTTCGTTCTTTCGTC	519
	MMP19-P1-R	C*AAGCTT*GAGTTTGTGCCCTCCGCTCT	

^a^*GAATTC*: EcoRI site

^b^*CTCGAG*: XhoI site

^c^*GGTACC*: KpnI site

^d^*AAGCTT*: HindIII site

### Western Blot

The coding potential of the three POU2F3 isoform expression plasmids (pCMV-Myc-POU2F3-1, pCMV-Myc-POU2F3-2, and pCMV-Myc-POU2F3-3) was verified by western blot. Briefly, HEK293 cells were seeded on 6-well plates with 1.2 × 10^6^ cells/well. After overnight culture, the cells were transfected with pCMV-Myc-POU2F3-1, pCMV-Myc-POU2F3-2, or pCMV-Myc-POU2F3-3 using Lipofectamine 2000 reagent (Invitrogen). At 72 h after transfection, cells were washed twice with ice-cold PBS and lysed in RIPA Lysis Buffer (Beyotime) containing 10 μg/mL PMSF (Beyotime) for 30 min on ice. Then, the lysates were collected and centrifuged in Eppendorf tubes at 4 °C for 15 min. The equal amounts of protein from the cell lysates were re-suspended in gel sample buffer, separated by 10% SDS–polyacrylamide gel electrophoresis, and transferred to nitrocellulose membranes. The blots were blocked in PBS containing 5% (w/v) dry milk and 0.1% Tween 20 for 2 h and then incubated with primary antibody dilution buffer (Beyotime) containing Myc-tag mouse monoclonal antibody (Abcam, 1:1000) at room temperature for 2 h. After washing with PBS three times, the blots were incubated with a secondary antibody dilution buffer containing horseradish peroxidase-conjugated secondary antibody (1:5000) for 1 h, followed by washing with PBS for three times. Protein bands were visualized by chemiluminescence using the ECL kit (Sambrook et al. 1982).

### Cell Proliferation Assay

Cell Counting Kit-8 (CCK-8) was used to measure cell proliferation. Briefly, 5 × 10^4^ SFFs or HaCaT cells were seeded in 96-well plates and transfected with pCMV-Myc-POU2F3-1, pCMV-Myc-POU2F3-2, pCMV-Myc-POU2F3-3, or pCMV-Myc using Lipofectamine 2000 reagent (Invitrogen). At 24, 48, and 72 h after transfection, each well was added with 10 μL CCK-8 solution and incubated at 37 °C for 2 h, and then the absorbance was recorded at 450 nm.

### Dual Luciferase Reporter Assay

Dual luciferase reporter assay was performed in HEK293 cells. Briefly, 2.5 × 10^5^ cells were plated in 24-well plates, and co-transfected with the constructed POU2F3 expression vectors (pCMV-Myc-POU2F3-1, pCMV-Myc-POU2F3-2, pCMV-Myc-POU2F3-3, or pCMV-Myc) and the constructed promoter reporters (pGL3-basic-pKRT14 (− 699/ − 38) or pGL3-basic-pMMP19 (− 542/ − 24) together with pRL-TK (Promega) using Lipofectamine 2000 reagent (Invitrogen). At 48 h of transfection, the relative luciferase activity was determined using the Dual-Glo Luciferase Assay System (Promega). Relative luciferase activity was obtained by the normalization to Renilla luciferase activity, known as the ratio of Firefly to Renilla luciferase. All luciferase assays were performed in triplicate. The data represented at least three independent experiments.

### Statistical Analysis

All data were analyzed by ANOVA using the SPSS 19.0 software. *P* < 0.05 and *P* < 0.01 were considered statistically significant.

## Results

### Tissue Expression of POU2F3 Transcript Variants in Sheep

**Fig. 1:**
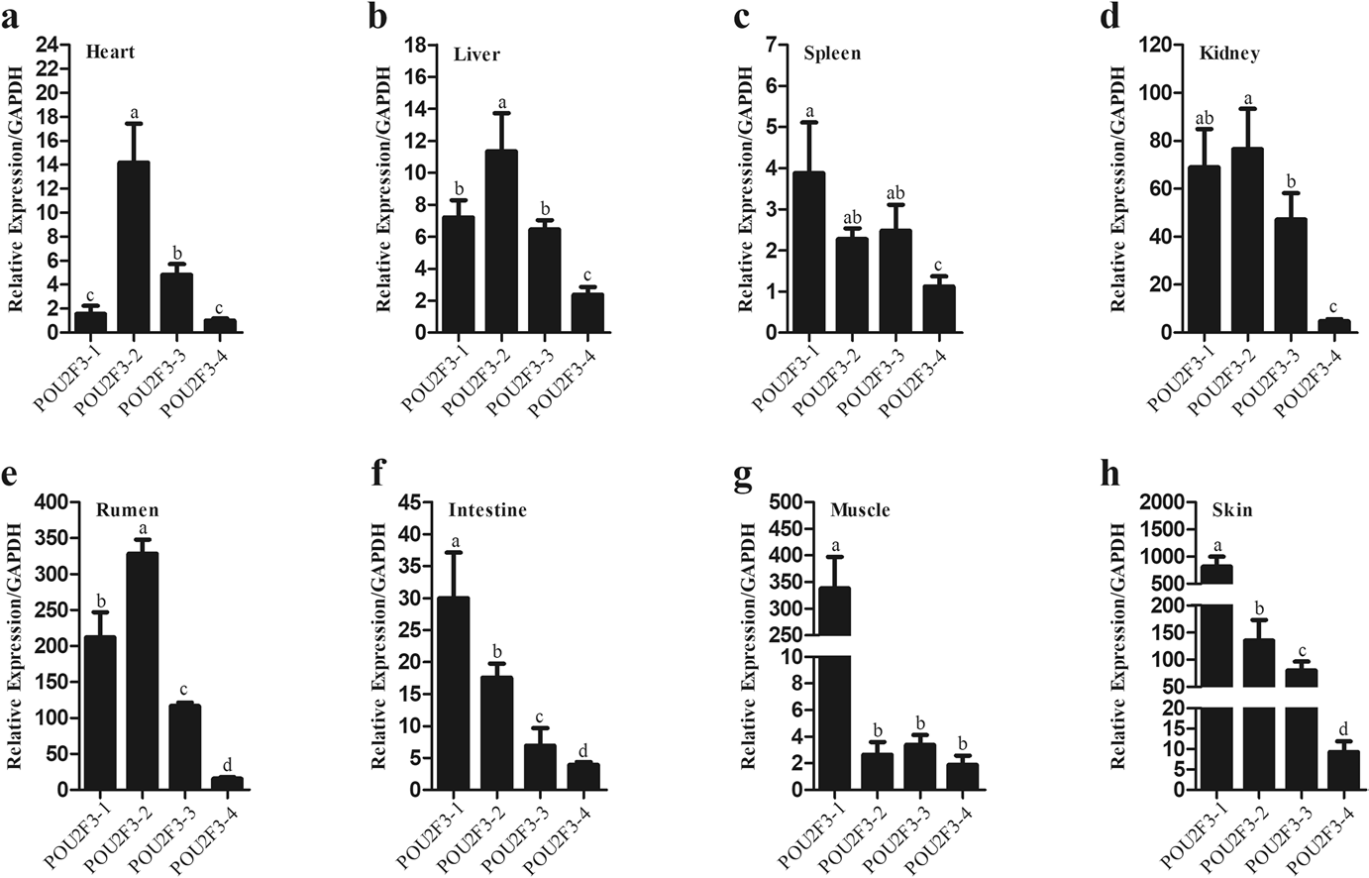
The expression analysis of four *POU2F3* transcript variants in adult sheep tissues. **a** Heart; **b** liver; **c** spleen; **d** kidney; **e** rumen; **f** small intestine; **g** skeletal muscle; **h** skin. The housekeeping gene *GAPDH* was used as an internal control for quantitative RT-PCR assay. Fold change was relative to mRNA expression of *POU2F3-4* in the heart. All data are representative of three independent experiments and are shown as the mean ± SEM. For each figure panel, different letters above error bars indicate a statistically significant difference (*P* < 0.05)

Our previous study showed that sheep *POU2F3* gene produces four transcript variants (*POU2F3-1*, *POU2F3-2*, *POU2F3-3,* and *POU2F3-4*), encoding three POU2F3 protein isoforms (POU2F3-1, POU2F3-2, and POU2F3-3) (Rong et al. 2013). Of these four sheep *POU2F3* transcript variants, *POU2F3-1* contained all exons, but *POU2F3-2* lacked exon 3, and *POU2F3-3* lacked exons 4 and 5, and *POU2F3-4* lacked exon 6 (Fig. S1). Based on their sequence characteristics, the transcript variant-specific primers were designed to detect tissue expression patterns of the four *POU2F3* transcript variants in sheep using quantitative RT-PCR. The results showed that the four *POU2F3* transcript variants were ubiquitously and differentially expressed in all tested adult sheep tissues including heart, liver, spleen, kidney, rumen, small intestine, skeletal muscle, and skin (Fig. 1). In the majority of the tested tissues, *POU2F3-1* and *POU2F3-2* were highly expressed, POU2F3-3 was intermediately expressed, and *POU2F3-4* was low expressed*.* These four *POU2F3* transcript variants were differentially expressed in the skin, and the expression levels of *POU2F3-1*, *POU2F3-2* and *POU2F3-3* were 87.60-, 14.54-, and 8.65-fold higher, respectively, than that of *POU2F3-4* (*P* < 0.05; Fig. 1h).

### Effects of Overexpression of POU2F3 Protein Isoforms on Cell Proliferation

**Fig. 2:**
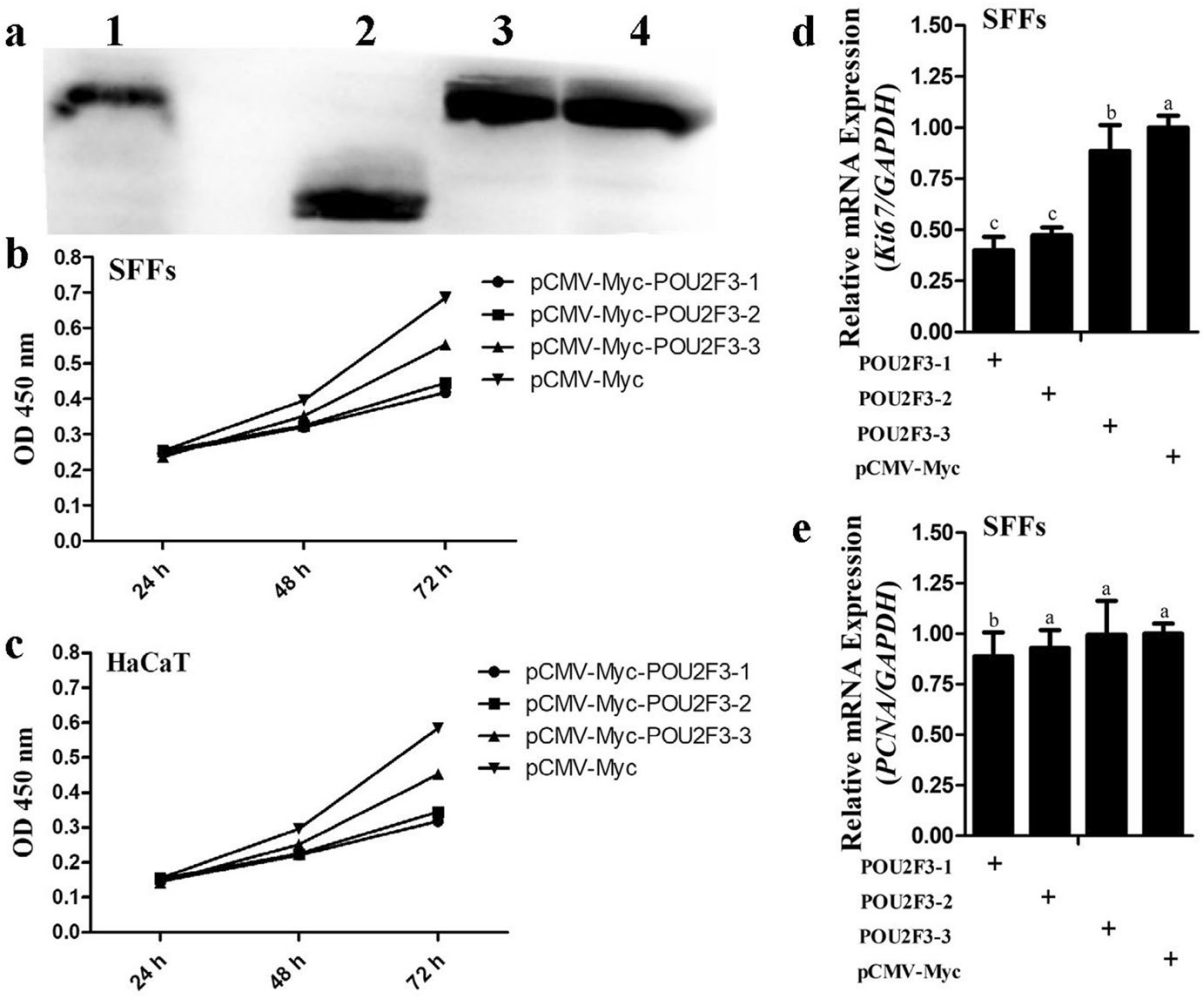
Effects of sheep POU2F3 isoforms on cell proliferation. **a** Western blot identification of three POU2F3 isoform expression vectors. Lane 1: positive control for Myc antibody (pCMV-Myc-FST, 38.2 kDa); lane 2: lysate of the cells transfected with pCMV-Myc-POU2F3-3 (27.7 kDa); lane 3: lysate of the cells transfected with pCMV-Myc-POU2F3-2 (43.3 kDa); lane 4: lysate of the cells transfected with pCMV-Myc-POU2F3-1 (46.9 kDa). **b**, **c** Effect of the three POU2F3 isoforms on the proliferation of SFFs (**b**) and HaCaT cells (**c**). 5 × 10^4^ cells were seeded in 96-well plates, and transfected with the indicated POU2F3 isoform expression vectors (pCMV-Myc-POU2F3-1, pCMV-Myc-POU2F3-2, and pCMV-Myc-POU2F3-3) or empty vector pCMV-Myc (0.8 μg/well), and cell proliferation was assayed at indicated time points after transfection using CCK-8 kit. **d**, **e** Quantitative RT-PCR assay of *Ki67* (**d**) and *PCNA* (**e**) at 48 h of transfection in SFFs cells. 1.2 × 10^6^ cells were seeded in 6-well plates, and transfected with the indicated POU2F3 isoform expression vectors or empty vector pCMV-Myc (4.0 μg/well), and at 48 h of transfection, total RNA was isolated from the cells with Trizol reagent, and gene expression was assessed using quantitative RT-PCR. The housekeeping gene *GAPDH* was used as an internal control for quantitative RT-PCR. Fold change was relative to the expression of the cells transfected with empty vector pCMV-Myc at 48 h of transfection. All data are representative of three independent experiments and are shown as the mean ± SEM. For each figure panel, different letters above error bars indicate a statistically significant difference (*P* < 0.05)

It had been reported that human POU2F3 could promote keratinocyte proliferation (Hildesheim et al. 2001), however, the knockout mice study (Andersen et al. 1997) and the colony inhibition assay in cervical cancer cells (Yutaka et al. 2004) suggested POU2F3 might inhibit keratinocyte proliferation. To investigate whether sheep POU2F3 regulates cell proliferation and whether these three sheep POU2F3 isoforms have different effects on cell proliferation, we constructed the three POU2F3 isoform expression vectors (pCMV-Myc-POU2F3-1, pCMV-Myc-POU2F3-2, and pCMV-Myc-POU2F3-3) and confirmed their expressions by western blot (Fig. 2a). These POU2F3 isoform expression plasmids were transiently transfected into either SFFs or HaCaT cells, and cell proliferation was assayed using CCK-8 assay. The results showed that the absorbance of both the SFFs and HaCaT cells transfected with any one of these POU2F3 isoform expression plasmids was significantly lower than that of the cells transfected with the empty vector pCMV-Myc at 48 and 72 h of transfection (*P* < 0.05, Fig. 2b, c), suggesting that overexpression of any one of the three POU2F3 isoforms inhibits the proliferation of SFFs and HaCaT cells. POU2F3-1 and POU2F3-2 overexpression had a similar inhibitory effect on the cell proliferation (*P* > 0.05, Fig. 2b, c), and both had greater inhibitory effects on the cell proliferation than POU2F3-3 in both SFFs and HaCaT cells at 72 h of transfection (*P* < 0.05, Fig. 2b, c).

In parallel, we detected the expression of proliferation marker genes (*Ki67* and *PCNA*) using quantitative RT-PCR. Consistent with CCK-8 results, overexpression of any one of POU2F3 isoforms in SFFs cells, significantly inhibited *Ki67* expression compared with the empty vector pCMV-Myc at 48 h of transfection (*P* < 0.05, Fig. 2d). POU2F3-1 and POU2F3-2 overexpression had similar inhibitory effects on the *Ki67* expression (*P* > 0.05, Fig. 2d), and both had greater inhibitory effects on the *Ki67* expression than POU2F3-3 overexpression (*P* < 0.05, Fig. 2d). Similarly, *PCNA* expression was decreased when cells were transfected with each one of three POU2F3 isoform expression vectors compared with the pCMV-Myc control (Fig. 2e). Taken together, these data suggested that these sheep POU2F3 isoforms inhibit cell proliferation, but to a different extent.

### Effects of Overexpression of POU2F3 Protein Isoforms on the Promoter Activity of KRT14 and MMP19 Genes

**Fig. 3:**
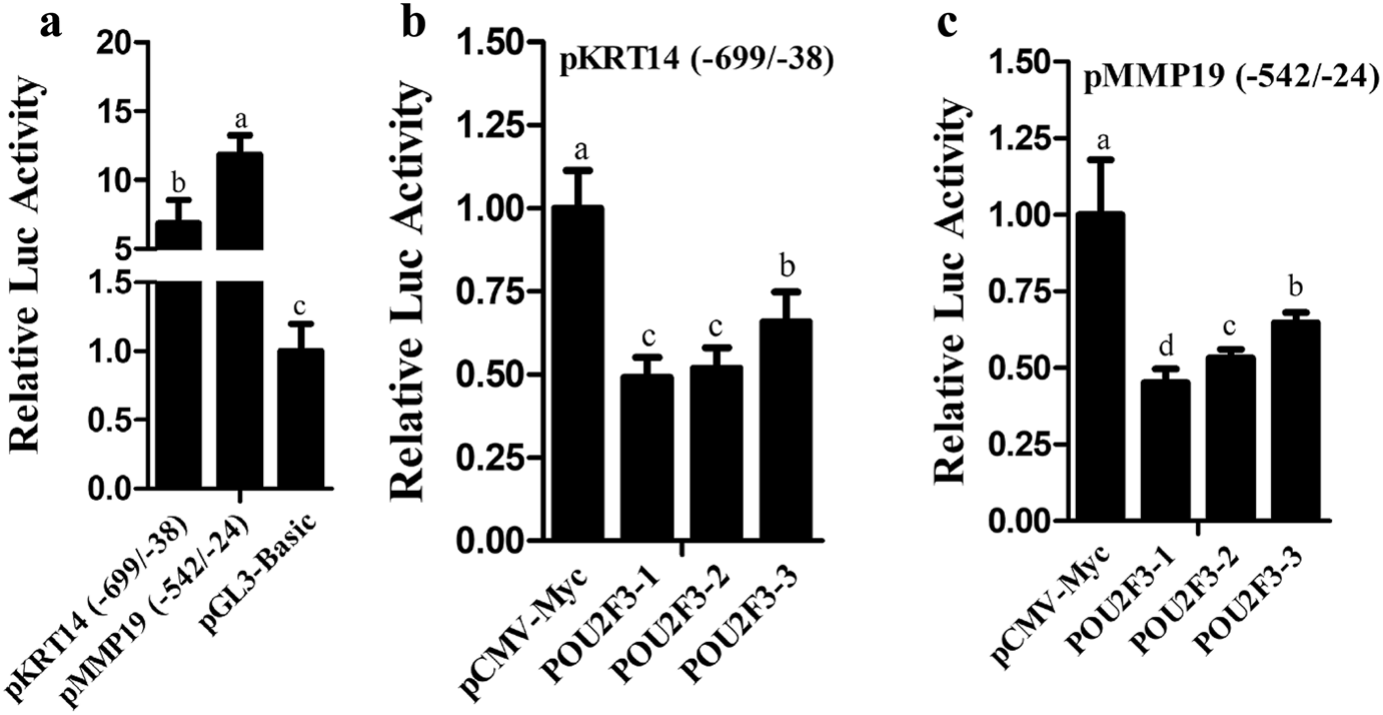
Effects of three sheep POU2F3 isoforms on the promoter activities of *KRT14* and *MMP19* genes. **a** Luciferase reporter assay of sheep *KRT14* promoter (− 699/ − 38) and *MMP19* promoter (− 542/ − 24) in HEK293 cells at 48 h of transfection. Fold change was relative to the pGL3-basic at 48 h of transfection. **b**, **c** Effects of the three sheep POU2F3 isoforms on the promoter activities of *KRT14* and *MMP19* genes. Either of pGL3-basic-pKRT14 (− 699/ − 38) or pGL3-basic-pMMP19 (− 542/ − 24) and the indicated isoform expression vectors (pCMV-Myc-POU2F3-1, pCMV-Myc-POU2F3-2, or pCMV-Myc-POU2F3-3) were co-transfected into HEK293 cells. Forty-eight hours after transfection, cells were lysed and luciferase activity was measured. Results were normalized with the Renilla luciferase activity, and expressed as the ratio of Firefly to Renilla luciferase. Fold change was relative to the empty vector pCMV-Myc at 48 h of transfection. All data are representative of three independent experiments and are shown as the mean ± SEM. For each figure panel, different letters above error bars indicate a statistically significant difference (*P* < 0.05)

Previous studies have demonstrated that POU2F3 down-regulated the promoter activities of *KRT14* and *MMP19* genes in HaCaT and HEK293 cells (IM et al. 2007; Sugihara et al. 2001). To reveal the functional differences among the three POU2F3 isoforms, we also investigated their effects on the promoter activity of *KRT14* and *MMP19* genes. The reporter gene assays showed that the cloned *KRT14* and *MMP19* promoters (pGL3-basic-pKRT14 (− 699/ − 38) and pGL3-basic-pMMP19 (− 542/ − 24)) were active compared with pGL3-basic vector (*P* < 0.05, Fig. 3a), and transfection of any one of the three POU2F3 isoform expression vectors significantly inhibited the promoter activities of *KRT14* and *MMP19* genes compared with the empty vector pCMV-Myc (*P* < 0.05, Fig. 3b, c). For *KRT14* promoter, POU2F3-1 and POU2F3-2 showed similar inhibitory effects (*P* > 0.05, Fig. 3b), and they had greater inhibitory effects on the *KRT14* promoter activity than POU2F3-3 (*P* < 0.05, Fig. 3b). For *MMP19* promoter, POU2F3-1 had a greater inhibitory effect than POU2F3-2 and POU2F3-3 (*P* < 0.05, Fig. 3c), and POU2F3-2 had a greater inhibitory effect than POU2F3-3 (*P* < 0.05, Fig. 3c).Taken together, these data suggested that three sheep POU2F3 isoforms inhibit the promoter activities of *KRT14* and *MMP19* genes, but to a different extent.

## Discussion

Alternative splicing is a crucial mechanism of regulation of gene expression and protein diversity (Barmak and Christopher 2001; Stamm et al. 2013). High-throughput sequencing has revealed that 92–94% of human genes undergo alternative splicing (Qun et al. 2008; Wang et al. 2008). Alternative splicing generates protein isoforms with different or even opposite functions (Stamm et al. 2013). Our previous finding showed that sheep *POU2F3* produces four mRNA transcript variants due to alternative splicing, and encodes three protein isoforms (POU2F3-1, POU2F3-2, and POU2F3-3) with various N terminus (Rong et al. 2013). In the present study, our results showed that four sheep *POU2F3* transcript variants were widely and differentially expressed in various tissues of the Chinese Merino sheep, especially the skin (Fig. 1), suggesting that POU2F3 alternative splicing is regulated in a tissue-specific manner and that these POU2F3 isoforms might exert different functions. Consistently, we found that these three sheep POU2F3 protein isoforms inhibited the proliferation of SFFs and HaCaT cells, but to a different extent (Fig. 2b, c).

In agreement with our results, several reports have indicated that POU2F3 inhibits cell proliferation (Andersen et al. 1997; Sadowski et al. 2003; Sugihara et al. 2001; Yutaka et al. 2004). However, human POU2F3 has been shown to promote keratinocyte proliferation (Hildesheim et al. 2001). This difference suggests that POU2F3 may play different roles in cell proliferation, depending on cell type, cellular context, and species.

*KRT14* and *MMP19* genes are two target genes of POU2F3, highly expressed in mitotically active epithelial basal cells, and their expressions are down-regulated during cell differentiation (Beck et al. 2007; Sugihara et al. 2001). In HaCaT cells, *KRT14* knockdown inhibited cell proliferation (Alam et al. 2011), while *MMP19* overexpression increased cell proliferation (IM et al. 2007). These data indicate that *KRT14* and *MMP19* are proliferation-promoting genes. Consistent with this notion, our results showed that the three sheep POU2F3 isoforms inhibited the promoter activities of *KRT14* and *MMP19* genes (Fig. 3b, c) and the proliferation of SFFs and HaCaT cells to a different extent (Fig. 2b, c). Combined with these data, it is reasonable to speculate that sheep POU2F3 might inhibit cell proliferation by down-regulating *KRT14* and *MMP19* genes.

The POU class of transcription factors is characterized by the POU-specific domain located in the upstream of a POU-homeodomain (Bürglin and Affolter 2016). POU-homeodomain is responsible for DNA-binding (Klemm et al. 1994) and protein–protein interaction (Cabral et al. 2003). The POU-specific domain is also involved in DNA-binding. This domain binds to DNA in two conformations (Reményi et al. 2001). In one conformation, POU-specific domain binds to a 15-bp DNA sequence called PORE, which is not palindromic. In another conformation, it binds to a 12-bp palindromic DNA sequence, named MORE (Bürglin and Affolter 2016). The previous study showed that hSkn-1a containing all coding exons with amino acids of 1–430 repressed the *hKRT14* promoter activity by about threefold in normal human epidermal keratinocytes (*P* < 0.05) (Sugihara et al. 2001). Interestingly, △C-hSkn-1a lacking C-terminal repressed the *hKRT14* promoter activity to the same extent as hSkn-1a (*P* > 0.05), however, △N-hSkn-1a removing N-terminal significantly decreased the inhibitory effect compared with hSkn-1a (*P* < 0.05) (Sugihara et al. 2001). These results suggest that the POU-specific domain and POU-homeodomain are required for the inhibitory effect of hSkn-1a and ΔC-hSkn-1a on the *hKRT14* promoter activity, and the full repression requires the complete N-terminal. Consistent with this suggestion, our study showed that all these three sheep POU2F3 isoforms inhibited the promoter activities of *KRT14* and *MMP19* genes to a different extent. Their inhibitory effects might be explained by their shared POU-homeodomain (Rong et al. 2013), and the extent of different inhibition might be due to their differences in the N-terminal and POU-specific domain (Rong et al. 2013). It is worth investigating the molecular mechanisms underlying the functional differences among the three POU2F3 isoforms in the future.

## Conclusion

This is the first report showing the functional differences among sheep POU2F3 isoforms. We demonstrated that the four sheep *POU2F3* transcripts were ubiquitously and differentially expressed in adult Chinese Merino sheep tissues, and the three sheep POU2F3 protein isoforms inhibited the proliferation of SFFs and HaCaT cells and the promoter activities of *KRT14* and *MMP19* to a different extent.

## Electronic supplementary material

Below is the link to the electronic supplementary material.

Supplementary file1 (DOCX 503 kb)

Supplementary file2 (XLSX 20 kb)
