# Supplementary material for: Functional Analysis of Sheep POU2F3 Isoforms
Source: Biochem Genet. 2019 Dec 31;58(2):335–47. doi: 10.1007/s10528-019-09945-x (PMC7113193; doi:10.1007/s10528-019-09945-x)
Supplement: Supplementary file 1 — Supplementary file1 (DOCX 503 kb) [file 10528_2019_9945_MOESM1_ESM.docx]

**Supplementary Materials**

**Fig.** **S1.** Genomic structure and alternative splicing of sheep *POU2F3* gene. Exons are indicated by filled boxes, the skipped exons are indicated by open boxes, introns are indicated by lines, and numbered from 1 to 12. Thick arrows denote the transcript-specific primers used for the expression analysis of *POU2F3* transcript variants in sheep. F1 and R1: POU2F3-1-P1-F and POU2F3-1-P1-R; F2 and R2: POU2F3-2-P1-F and POU2F3-2-P1-R; F3 and R3: POU2F3-3-P1-F and POU2F3-3-P1-R; F4 and R4: POU2F3-4-P1-F and POU2F3-4-P1-R
